# Aging of 3D Printed Polymers under Sterilizing UV-C Radiation

**DOI:** 10.3390/polym13244467

**Published:** 2021-12-20

**Authors:** Catalin Gheorghe Amza, Aurelian Zapciu, Florin Baciu, Mihai Ion Vasile, Diana Popescu

**Affiliations:** 1 Department of Quality Engineering and Industrial Technologies, Faculty of Industrial Engineering and Robotics, University Politehnica of Bucharest, 060042 Bucharest, Romania; vasileionmihai@yahoo.com; 2 Department of Robotics and Production Systems, Faculty of Industrial Engineering and Robotics, University Politehnica of Bucharest, 060042 Bucharest, Romania; aurelianzapciu@yahoo.com (A.Z.); diana.popescu@upb.ro (D.P.); 3 Department of Strength Materials, Faculty of Industrial Engineering and Robotics, University Politehnica of Bucharest, 060042 Bucharest, Romania; florin.baciu@upb.ro

**Keywords:** 3D-printing, PLA, PETG, accelerated aging, ultraviolet, UV-C

## Abstract

In the context of the COVID-19 pandemic, shortwave ultraviolet radiation with wavelengths between 200 nm and 280 nm (UV-C) is seeing increased usage in the sterilization of medical equipment, appliances, and spaces due to its antimicrobial effect. During the first weeks of the pandemic, healthcare facilities experienced a shortage of personal protective equipment. This led to hospital technicians, private companies, and even members of the public to resort to 3D printing in order to produce fast, on-demand resources. This paper analyzes the effect of accelerated aging through prolonged exposure to UV-C on mechanical properties of parts 3D printed by material extrusion (MEX) from common polymers, such as polylactic acid (PLA) and polyethylene terephthalate-glycol (PETG). Samples 3D printed from these materials went through a 24-h UV-C exposure aging cycle and were then tested versus a control group for changes in mechanical properties. Both tensile and compressive strength were determined, as well as changes in material creep properties. Prolonged UV-C exposure reduced the mechanical properties of PLA by 6–8% and of PETG by over 30%. These findings are of practical importance for those interested in producing functional MEX parts intended to be sterilized using UV-C. Scanning electron microscopy (SEM) was performed in order to assess any changes in material structure.

## 1. Introduction

In the early weeks of the COVID-19 pandemic, emergency rooms, hospitals, clinics, and other healthcare facilities worldwide experienced a shortage of personal protective equipment (PPE), as signaled at the time by the World Health Organization (WHO) [1] and since then by many other reports and studies [2,3,4,5,6]. The decentralized production achievable by 3D printing [7,8] has allowed hospital technicians, companies, or even members of the public to rapidly manufacture components and consumables that can replace medical equipment in the case of a supply shortage [9,10].

While some of these products, such as sterile nasopharyngeal swabs [11,12], are meant for single use, other parts such as face shields can be reused if properly sterilized [13,14,15]. While 3D printing allows parts to be manufactured on-demand and on-site or near-location, reducing the time and overhead until personnel can use the finished product, this manufacturing method can have other advantages as well. Studies revealed that N95 respirator efficiency in filtering particles and preventing infection with a virus such as SARS-CoV-2 increases when fitted to obtain a tight seal between the mask and the user’s face [16,17,18]. As 3D printing is suitable for manufacturing personalized items, Ballard et al. 3D printed N95 alternative masks designed based on digital facial topography data. These prototypes passed OSHA-certified quantitative respiratory testing [19]. Another implementation is that of McAvoy et al., who 3D printed frames that can attach to commercial N95 and KN95 respirators, improving their fit [20].

Common sterilization methods of medical equipment include steaming (autoclaving), dry heat treating, ultrasonic sterilization, or exposure to agents, such as hydrogen peroxide or ethylene oxide [21,22,23,24,25]. Work by Grzelak et al., on the effects of alcohol disinfection on ABS and PETG parts showed that PETG parts can lose 20% of their tensile strength after exposure to sterilizing alcohol [26]. In a study by Popescu et al. [27], repeated hydrogen peroxide vapor and low temperature gas plasma sterilization were studied, as autoclaving the 3D printed acrylonitrile butadiene styrene parts deforms the material. Thus, it is expected that the steam sterilization method cannot be applied to materials with lower thermal resistance, such as polylactic acid (PLA) or polyethylene terephthalate-glycol (PETG). Indeed, a review by Davila et al. highlights the difficulties encountered when sterilizing PLA parts [28].

During the COVID-19 pandemic, interest in the germicidal action of UV-C [29,30,31,32] has increased considerably. While UV-A and UV-B radiation are produced by the Sun and reach Earth’s troposphere naturally, UV-C is artificially created. For this reason, UV-A and UV-B have well defined accelerated aging testing standards [33] as part of the methods used for the accelerated aging of plastics, which include methods such as open-flame or xenon-arc, while UV-C testing standards are not well defined and regulation surrounding the use of this technology is sparse at this moment. The disparity found in standardization is also found in studies on the effects of various radiation types, with the majority of available research information focusing on UV-A and UV-B effects. While studying the biodegradability of PLA composites, Yu et al. found that short-term UV exposure increases the tensile properties of the investigated materials, while long term exposure has an increasingly detrimental effect [34].

Effects of other types of radiation on polymers have also been investigated. Chaochanchaikul and Harnnarongchai [35] showed that gamma irradiation decreases the weight-averaged molecular weight and number-averaged molecular weight in PLA material. Cairns et al. showed that the changes in the molecular weights of PLA are dependent on the amount of radiation exposure [36]. Nugroho et al. [37] noted that chain degradation in PLA can occur during gamma irradiation in air and vacuum. The same paper indicated that, with an increase in radiation strength, the polymer is more susceptible to oxidative chain-scission.

Following a January 2020 joint workshop between the International UV Association (IUVA) and the United States’ National Institute of Standards and Technology (NIST) [38], a Special Section on Ultraviolet Technologies for Public Health was opened to submissions on this topic, seeking to provide guidance for new applications of UV-C technology and for new methods to characterize the effects of such radiation on physical and biological materials [39]. Answering the call, Poster et al., wrote about the requirements in this field for standards and metrology [40], while Kreitenberg and Martinello proposed several recommendations regarding whole-room disinfection using UV-C radiation [41]. As of today, such standards are currently under development as part of a collaboration between the NIST and the Illuminating Engineering Society (IES.org).

Iriving et al., studied the degradation caused by UV-C sterilization on flexible endoscopes and concluded that 405 nm radiation is a safer alternative [42]. Geldert et al. studied the effects of UV-C on N95 respirators [43]. Copinet et al., studied the effects of 315 nm radiation (at the border between UV-A and UV-B spectrums) on PLA films in various atmospheric conditions and concluded that UV radiation accelerates degradation [44]. Zhang et al., highlighted the effects of UV-C irradiation on PLA, also concluding that UV accelerates degradation process and that the molecular weight loss increases linearly with exposure time [45]. For PETG, Arangdad et al., studied the impact of UV exposure on degradation and the influence of various UV photostabilizers in reducing this degradation [46].

Other than knowing the ultimate tensile and compressive strength, in real world applications it is important to understand the creep behavior of materials [47]. Creep is the tendency of certain plastics and fibers to deform plastically, over time, under stress significantly lower than the one identified at ultimate strength. Scientific literature proves that both PLA [48,49,50] and PETG [51,52] experience tensile and compression creep. In this area, Tezel et al., found that part infill orientation changes the creep behavior of PLA 3D printed parts and concluded that a 90° orientation is the most beneficial in reducing creep [53]. Other research groups, such as Mohammadizadeh et al., showed the effect of reinforcing fibers on improving creep behavior of 3D printed nylon parts [54]. Regarding the effects of UV on creep behavior, Romeijn et al., pointed to UV light as being the only environmental stressor within a series of investigated effects to increase the creep compliance of 3D printed PETG [51]. An example of a part where creep plays an important role in terms of usability is the 3D printed band for a protective face shield [13].

Thus, one of the objectives of this research was also to assess the changes in creep behavior after the accelerated aging process.

Considering the importance of analyzing the effect of UV-C and the expansion of the use of 3D printing, the need for further investigation of the interaction of UV-C with materials is warranted. In this context, this paper intends to provide greater insight into the changes in mechanical properties suffered by 3D-printed parts made from two widely available 3D-printing materials (PLA-polylactic acid and PETG-polyethylene terephthalate, glycol-modified) after prolonged exposure to artificial UV-C radiation. By replacing the prolonged exposure with a controlled irradiation treatment, the same damaging effect can be obtained in a much shorter time. Among the effects investigated in this paper are the loss in tensile and compression strength following irradiation, part stiffness, and tensile and compression creep behavior changes. By determining the amount of degradation of mechanical properties, part designers can create parts that withstand repeated sterilizations.

## 2. Materials and Methods

### 2.1. Sample 3D Printing and UV-C Exposure Protocol

**Figure 1:**
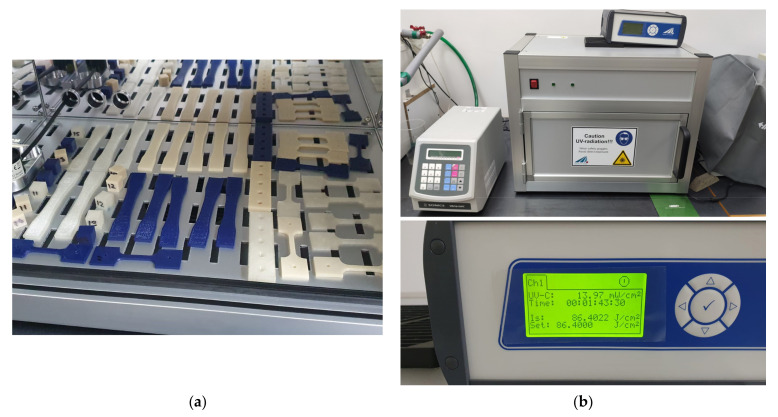
Tested materials (**a**) 3D printed parts; (**b**) UV-C exposure of tested groups.

As mentioned in Section 1, standards for UV-C accelerated aging are not well defined. An appendix of the ISO 4892-2 standard mentions the possibility to modify standard testing conditions to allow the usage of a mercury lamp that generates 10 W/m^2^ of 254 nm wavelength radiation. Another testing protocol is found in documentation provided by the Business and Institutional Furniture Manufacturers Association (BIFMA) for healthcare furniture design and specifies a sample should be subjected to a radiative energy of 291 kJ/m^2^ between 12 and 24 h [55]. However, these are not specific to the medical industry. For the purpose of this study, an irradiation power of 10 W/m^2^ and a backpanel temperature of 50 °C were used. The 3D printed samples (Figure 1a) were subjected to UV-C irradiation (Figure 1b) for 24 h and then left to cool down to room temperature for 4 h. The equipment used to perform the accelerated aging process is a BS-02 irradiation chamber made by Opsytec Dr. Grobel (Ettlingen, Germany).

The effect of accelerated aging was assessed in compliance with the ISO 4892–1:2016 standard, which offers guidance on how to analyze the data resulting from artificial accelerated irradiation exposures [56]. Tests have been designed according to ISO 4582:2017 [57] and include tensile strength testing and compressive strength tests.

The materials analyzed in this study are two of the most common materials used with filament-based 3D printing, PLA (polylactic acid) and PETG (polyethylene terephthalate, glycol-modified). The PLA material used was PLA Extrafill filament (opaque, blue), 1.75 mm in diameter, manufactured by Fillamentum (Hulin, Czech Republic). For this material, the manufacturer specifies a glass transition temperature of 55–60 °C and a melting temperature of 140–165 °C. The other 3D printing material used for fabricating test specimens is PrimaSelect transparent PETG filament, 1.75 mm in diameter, manufactured by Prima Printer Nordic AB (Malmö, Sweden). For this material, the manufacturer specifies a glass transition temperature of 86 °C and a melting temperature of 255 °C. The slicer software used to section the 3D models into horizontal layers is Cura 4.5 (Ultimaker, Utrecht, The Netherlands). All specimens were 3D printed on a Creality Ender 3 3D printer (Shenzhen Creality 3D Technology, Shenzen, China). The stock 3D printer came equipped with a 0.4 mm nozzle, a size commonly used by other 3D printer manufacturers as well. For layer settings, a width equal to 100% of the nozzle diameter was used (0.40 mm) while layer height was set at 50% of the nozzle diameter (0.20 mm). In MEX, the number of outside perimeters (contours) influences the mechanical properties of the fabricated part due to the inherent anisotropic properties [58]. This effect is more pronounced in narrow parts, such as the samples fabricated for tensile tests [59]. Two contours were used in order to balance the influence of the outside perimeter with that of the infill. By using 100% infill with a grid pattern at a 45° raster angle, the performance of the tested samples should be closer to that of a typical functional part. Extrusion temperatures were selected based on manufacturer recommendations and were set at 205 °C for all PLA specimens and 235 °C for PETG. A heated bed was used, set at 45 °C for PLA and 65 °C for PETG. Printing process parameters are shown in Table 1.

For each of the test types (tensile strength, compressive strength, tensile creep, compression creep), one group of 5 specimens was subjected to accelerated aging using UV-C radiation using the parameters highlighted previously. The second group of 5 specimens formed the control group. Visual and dimensional inspections were performed before and after the aging treatment.

### 2.2. Tensile Strength

For tensile strength tests, 10 specimens were 3D printed from each material according to ASTM type I dimensions [60]. It is worth mentioning that work done by Laureto et al. shows a difference between type I and type IV samples when testing MEX/FDM (Material Extrusion/Fused Deposition Modelling) 3D printed parts [61]. The samples where then randomly divided into 2 groups consisting of 5 specimens each.

The 3D printed specimens were tested for tensile strength and stiffness on an Instron 8872 (Norwood, MA, USA) universal testing machine, at a loading speed of 1 mm/min and a preload force of 5 N. Elongation of the sample was measured using an electronic extensometer. The results of tensile strength and stiffness testing (stress-strain charts) are presented in Section 3.2.

### 2.3. Compressive Strength

For compressive strength tests, 10 cubic specimens of 15 mm × 15 mm × 15 mm in dimensions were 3D printed from each material. The samples where then randomly divided in 2 groups consisting of 5 specimens each.

Compressive strength testing of the cubic samples was done on an Instron 8801 (Norwood, MA, USA) universal testing machine. During testing, the compressive load was applied along the Z-axis of the cubic samples. A preload force of 5 N was used. The results of compressive strength testing are discussed in Section 3.3.

### 2.4. Creep Characteristics

For tensile creep testing, 10 specimens were 3D printed from each material, in accordance with methods found in standard ASTM D2990-17 [62]. The narrow section of the part is 5.2 mm × 3 mm.

**Figure 2:**
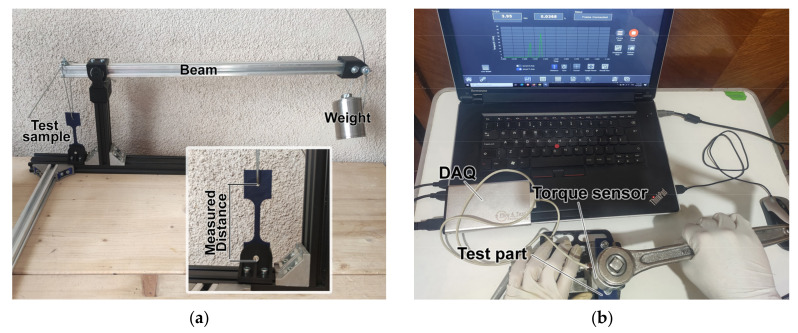
Creep properties testing: (**a**) Tension creep testing rig set at 9.4 × mechanical advantage and with 1250-g weight; (**b**) Compression creep testing: tightening to standard torque.

Tensile creep testing was performed by tensile loading 3D printed specimens with a load corresponding to 25% of the measured tensile strength for samples from the control group. Hence, 3D printed samples were placed in a purposefully designed rig with a variable length balance beam providing variable mechanical advantage (Figure 2a). One end of the beam was loaded with a known weight calculated based on tensile test results. Elongation of the specimen was measured after 2 h, 6 h, 12 h, and 24 h, then every 24 h for a total of 5 days.

For compressive creep testing, a more practically oriented method was chosen, as follows. A practical compressive creep test was performed by compression loading 3D printed specimens with a clamping force corresponding to the standard tightening torque for an M6 fastener (5.07 N·m). This was done by tightening a bolt and nut assembly on an 8 mm thick 3D printed plate. The clamping force was transmitted to the 3D printed plate through a metal washer with an outer diameter of 12 mm and an inner diameter of 7.5 mm. Tightening was performed using a wrench attached to a torque sensor, MR55-1000 (Mark-10 Corporation, Copiague, NY, USA). After 7 days, the bolt and nut assembly was undone, and the required untightening torque was measured using the same torque sensor (Figure 2b). Creep testing results are discussed in Section 3.4.

All mechanical tests described in this paper were performed at 24 °C and 50% humidity.

### 2.5. Scanning Electron Microscopy (SEM)

Scanning electron microscopy analysis (SEM) was performed in order to reveal any potential changes to material internal structure and to outer surfaces of the irradiated samples. For this analysis, samples were selected for investigation from each group and for each material (2 × PLA treated, 1 × PLA control, 2 × PETG treated, 1 × PETG control). The internal morphology of the samples was analyzed using a scanning electron microscope with 1.2 nm resolution (Quanta Inspect F50 from Thermo Fisher Scientific–Eindhoven, The Netherlands). Before imaging, all samples were sputter-coated with a thin layer of gold using a Q150 PlusSeries machine (Quorum Technologies, Lewes, UK). Sample coverage time was 90 s and the resolution of the energy-dispersive X-ray analyzer was set at 133 eV at MnKα. The results of this analysis are discussed in Section 3.5.

## 3. Results

### 3.1. Visual and Dimensional Changes Following UV-C Exposure

Visual and dimensional analyses were performed on the parts following the UV-C aging process. Table 2 presents dimensions of samples manufactured for creep testing, from both the irradiated and control groups, measured with electronic calipers. This ensured that a dimensional check was performed on both thin and long parts (tensile creep specimen, 5.2 mm × 3 mm section) and on bulky parts (compression creep specimen, 25.2 mm × 8 mm section). Measurements show there were no significant changes in part dimensions following the 24-h UV-C exposure cycle.

**Figure 3:**
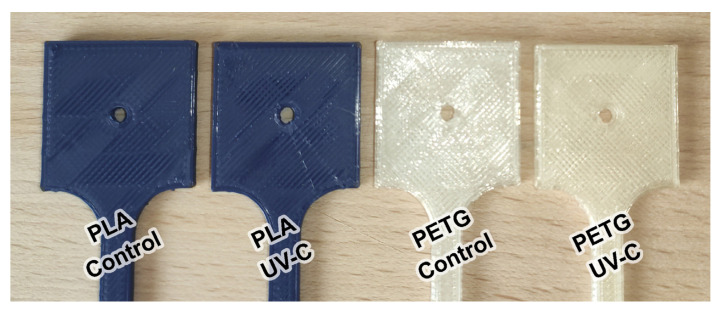
Changes in material color and reflectivity following UV-C exposure.

Figure 3 shows a visual comparison between the appearance of parts from the control group versus those from the UV-C exposed group. There were no visible changes in the appearance of PLA parts post exposure to ultraviolet radiation. The PETG samples darkened and gained a yellow tint. This was accompanied by a loss in reflectivity.

### 3.2. Tensile Strength and Young’s Modulus

**Figure 4:**
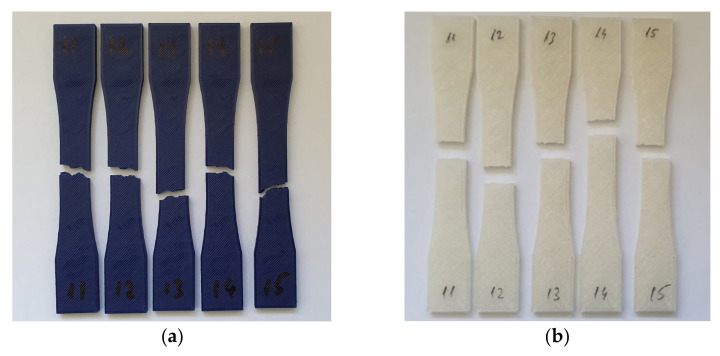
Fracture modes of tested parts: (**a**) Fractured PLA parts exposed to UV-C; (**b**) PETG parts exposed to UV-C.

The fracture mechanism for tensile tested specimens was visually analyzed and it was found that the PLA samples ruptured in a zig-zag pattern, along the deposited polymer filaments, for both the irradiated and control groups (Figure 4a). This failure mechanism was expected due to the anisotropic aspect of 3D printed parts where tensile strength along the deposited filaments is higher than that between adjacent filaments [63]. In MEX, a heated extruder brings a filament of material near its melting point and then it extrudes it through a nozzle. Immediately after exiting the nozzle, the material begins cooling through convective interaction with air. Once the extruded material comes into contact with the 3D printed part, it also transfers heat through conduction [64]. Because the convective effect is the dominant thermal transfer effect, the outer surface of the deposited filament cools down faster than the center, creating a temperature gradient [65]. This temperature gradient produces non-uniform properties in the 3D printed part, as the bonds formed between adjacent filaments are weaker than those found in the midsection of filaments. For PETG parts from both groups, fractures were focused along a single plane due to the better interlayer adhesion characteristic of this material (Figure 4b). The fact that both the control group and the UV-C exposed group experienced the same failure modes points to an isotropic degradation of the mechanical properties in the investigated samples.

**Figure 5:**
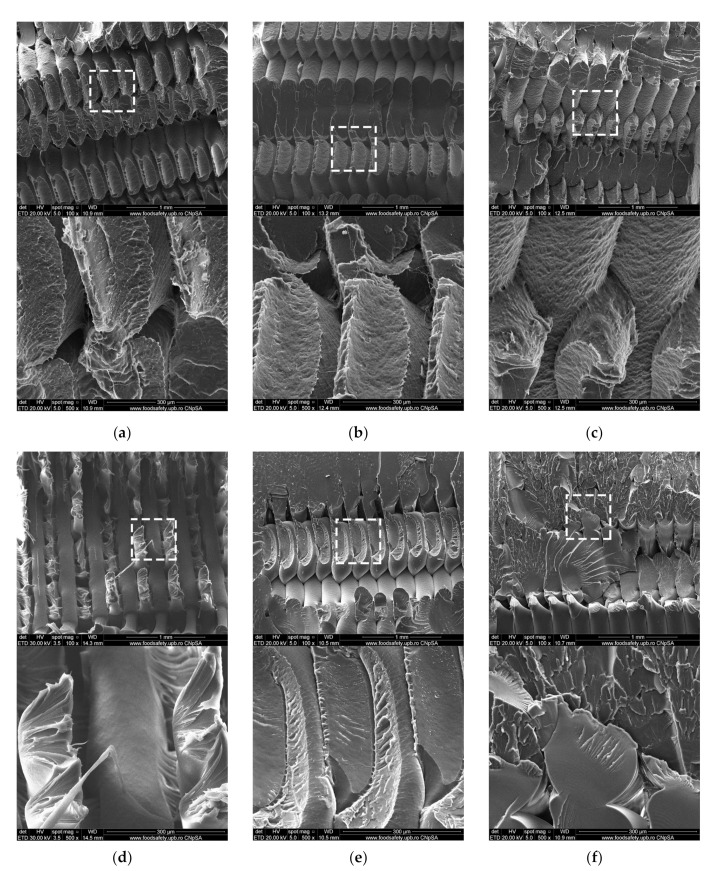
Tensile strength testing results, with Young’s Modulus and tensile stress at tensile strength: (**a**) PLA UV-C exposed group (solid) and control group (dashed); (**b**) PETG UV-C exposed group (solid) and control group (dashed).

Figure 5a shows the stress–strain diagrams obtained from the tensile testing of PLA, with the results for PETG being shown in Figure 5b.

Parts made of PLA sustained a 9.1% loss in tensile strength following UV-C exposure compared to those in the control group (26.86 MPa vs. 29.54 MPa, F = 28.53, *p* = 6.9 × 10^−4^). On average, the stiffness of the parts in the irradiated group saw a small decrease of 1.9% (2721 MPa vs. 2775 MPa). After calculating the standard error for results of each group, it was found that the observed decrease in stiffness is not statistically significant (F = 1.98, *p* = 0.19).

The samples made of PETG that were subjected to accelerated aging treatment experienced a significantly higher tensile strength loss, losing 38.1% of their strength compared to PETG parts in the control group (19.48 MPa vs. 31.30 MPa, F = 522.27, *p* = 1.4 × 10^−8^). The Young’s modulus of the irradiated PETG changed slightly to the downside, lowering by 5.8% (1552 MPa vs. 1648 MPa, F = 15.55, *p* = 4.3 × 10^−3^). Table 3 shows the average tensile strength and the Young’s modulus of the tested parts with standard error.

### 3.3. Compressive Strength

**Figure 6:**
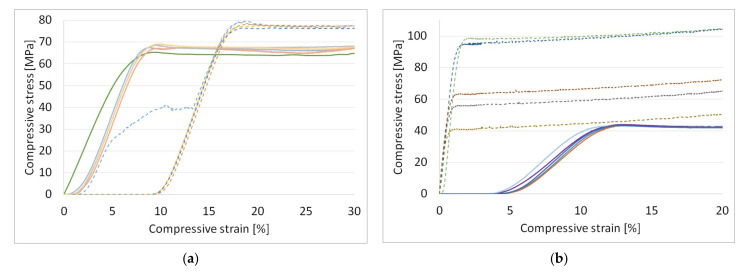
Compressive strength testing results: (**a**) PLA UV-C exposed group (solid) and control group (dashed); (**b**) PETG UV-C exposed group (solid) and control group (dashed).

During the compressive strength tests, all parts failed plastically without any visible shear penetrating the exterior contours. All the samples undergoing the accelerated aging process performed worse than their counterparts in the control groups. PLA samples lost 13.1% of their compressive strength following UV-C irradiation (67.69 MPa vs. 78.06 MPa, F = 142.73, *p* = 2.2 × 10^−6^) while PETG samples lost 33.9% of their compressive strength compared to the control group (43.57 MPa vs. 65.94 MPa, F = 6.17, *p* = 3.8 × 10^−2^). A stress–strain diagram for PLA is shown in Figure 6a while the same diagram for PETG is shown in Figure 6b.

Table 4 shows the average compressive strength found during testing compiled with standard errors.

### 3.4. Creep Characteristics of the Analyzed Materials

Following tensile testing, a 29.54 MPa tensile strength was found for PLA and a 31.3 MPa tensile strength was found for PETG. These values were reference points for selecting the loads applied during creep testing, with the creep stress level being set at 25% of the maximum tensile strength, i.e., 7.5 MPa stress induced in the PLA samples and 8 MPa stress induced in the PETG samples. The equivalent force loads for these levels of stress are shown in Table 5. In order to obtain these levels of stress, a mass of 1250 g was attached at one end of the beam of the testing rig, while the rotating support joint of the beam was positioned to produce a 9.4× force multiplication for PLA samples and a 10× multiplication for PETG samples.

The practical compression creep test was performed according to the conditions described in Section 2. The resulting stress from tightening the screw and nut assembly is proportional to the tightening torque. Considering a screw torque coefficient of 0.3, the resulting stress becomes 40.9 MPa, representing 52% of the compression strength of PLA and 62% of that of PETG.

**Figure 7:**
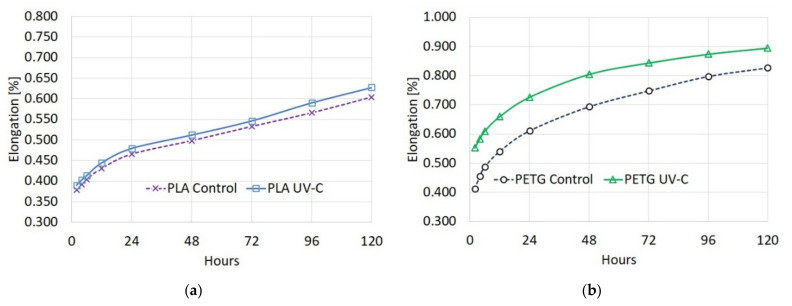
Creep curves of tested materials: (**a**) PLA elongation vs. time; (**b**) PETG elongation vs. time.

Part elongation was calculated after measuring the resulting deformation with a micrometer after 2 h, 6 h, 12 h, 24 h, 48 h, 72 h, 96 h, and 120 h. The graphs of elongation vs. time (Figure 7) show that UV-C did not have a significant impact on creep compliance. For both materials, the majority of the elongation happened immediately after stressing the parts with the tensile force. The elongation rate of change follows a relatively similar pattern for PLA parts after irradiation, consistent with the small changes in mechanical properties, especially part stiffness. For PETG parts, the elongation rate of change is slower after irradiation, a change that can be attributed to the deteriorated mechanical properties. Measurements are available in Supplementary Table S1.

**Figure 8:**
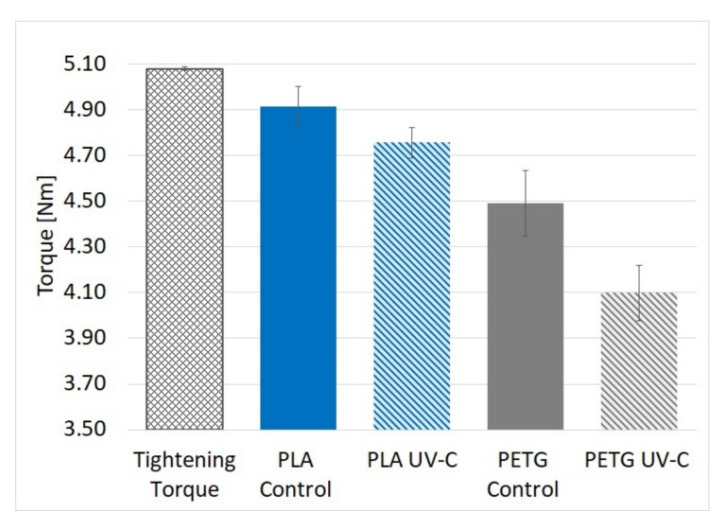
Results of compression creep practical test.

Following compression testing, it was found that all samples experienced a loss in clamping force after seven days, corresponding to a lower measurement of the untightening torque. The average torque reduction measured in PLA samples and attributed to UV-C radiation exposure was 3.2% (4.76 Nm vs. 4.92 Nm control group) and the largest reduction found in a single PLA sample was 5.2% (4.764 Nm vs. 5.031 Nm). For PETG, the average reduction was 8.8% (4.10 Nm vs. 4.49 Nm) and the largest reduction found in a single PETG sample was 9.6% (4.047 Nm vs. 4.478 Nm). The total reduction in torque was 6.4% for PLA and 23% for PETG. A chart of the untightening torque measurements is shown in Figure 8.

Average values for the untightening torque determined experimentally, with standard errors are shown in Table 6.

### 3.5. Scanning Electron Microscopy (SEM)

**Figure 9:**
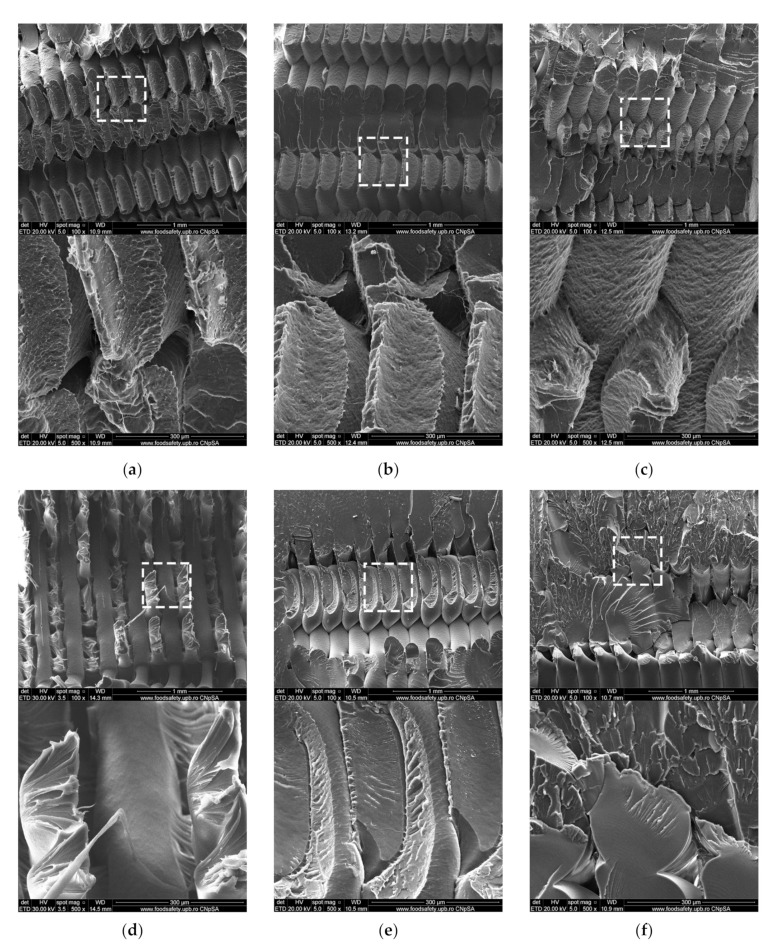
Scanning Electron Microscopy analysis of fractured 3D printed samples (**a**) PLA sample from control group (**b**) PLA sample from UV-C exposed group (**c**) PLA sample from UV-C exposed group; (**d**) PETG sample from control group; (**e**) PETG sample from UV-C exposed group; (**f**) PETG sample from UV-C exposed group.

SEM was used to get a better understanding of the changes occurring in the analyzed parts following the accelerated aging UV-C process. Figure 9a shows a ruptured PLA sample from the control group. As seen previously in the macroscale photographic images (Figure 4a), the sample ruptured along the deposited filaments, forming 45° fracture planes, indicative of 3D-printing specific anisotropy. Filament necking is visible at the fracture interface. In contrast, the first imaged PLA sample from the UV-C exposed group shows less material necking occurring (Figure 9b). As shown in Figure 9c the irradiated PLA sample shows increased roughness on the surfaces of deposited filaments. This change is not seen in the PETG samples and could be attributed to the material opacity and dark pigment.

PETG samples from both groups ruptured along a more uniform transversal plane, indicating a less pronounced anisotropic character, most likely due to increased interlayer and interfilament adhesion. Here, following irradiation, the reduction in filament necking at the fracture interface is more severe than in the case of PLA parts. The extensive necking found in a control group sample (Figure 9d) is replaced with a more defined fracture plane (Figure 9e) and with the appearance of material flaking at the rupture interface (Figure 9f). At microscale, less filament necking at the fracture interface and the development of flaking indicates lower elongation at break when tensile stressed. These changes point to an increase in the brittleness of the PETG samples after irradiation. This increased brittleness is also observed at a macroscale during tensile strength testing, as the tensile strength of PETG samples decreased by 38.1% while average elongation at break dropped from 3.06% to 1.36%. A probable cause for the reduction in mechanical properties of PETG is molecular photodegradation. Under the action of ultraviolet light, polymer materials experience chemical degradation through oxidative reactions [66]. It is suspected that the exposure to UV-C caused the photolysis of PETG, a chemical process whereby the transfer of light energy breaks the chemical bonds. Such changes have been identified by other researchers in PET (polyethylene terephthalate) [67]. All investigated samples show signs of microvoids specific to the fabrication process [68,69].

## 4. Discussion

In the context of the COVID-19 pandemic, the usage of 3D printing has seen an increase in the medical field, with healthcare facility technicians, businesses, or private individuals using this manufacturing process to try to alleviate some of the shortages in PPE and other medical resources. This brings forward an interest in finding out how 3D-printing materials behave in the conditions set forth by their novel use in the medical field. This paper analyzes the changes in mechanical properties of two commonly used polymers, PLA and PETG, after being subjected to sterilizing UV-C radiation. Test specimens were 3D printed from the above-mentioned materials and then randomly split into two group types: control groups and treatment groups. The treatment groups were subjected to a 24-h UV-C exposure cycle meant to simulate the effects of repeated UV-C sterilization procedures. Unlike other sterilizing methods, the UV-C aging cycles proved to not have any significant impact on part dimensions, regardless of part geometry and print orientation. No visual changes were observed following UV-C exposure other than darkening and yellowing of the transparent PETG samples.

Specimens made with ASTM D638-14 Type I dimensions were tested for tensile strength. Fracture modes were similar for control groups and aged groups. This indicates that irradiation produced mostly homogenous effects in the tested parts. Results showed a 9.1% tensile strength loss for irradiated PLA parts and a 38.1% loss for PETG parts. There was no significant change in the stiffness of PLA parts and a 5.1% reduction in Young’s Modulus for PETG parts.

Cubic test parts 3D printed from the two investigated materials were tested for compressive strength and similar losses in mechanical properties were found. PLA specimens showed a 45% reduction in compressive strength after UV-C aging while PETG test samples had a 38% reduction.

Test samples fabricated for tensile creep tests were tested in a rig made to exert a constant tensile force for a prolonged period of time (a total of 5 days). Elongation of the samples was measured at different intervals and elongation vs. time graphs were drawn. Experimental results point to minor changes in creep behavior that can be attributed to UV-C exposure. Most of the changes (elongation rate of change, elongation magnitude) can be attributed to the loss in tensile strength.

Bar specimens with holes were fabricated for compression creep tests. A screw and nut assembly was tightened to a standard tightening torque, exerting a clamping force on the tested part. After seven days, the assembly was untightened, and the required torque was measured using a torque sensor. The impact of UV-C radiation on the material creep compliance was a 3.2% reduction on PLA parts and 8.8% on PETG parts. These reductions in creep compliance are in addition to the clamping force losses of 3% for PLA and 11.5% for PETG found in control group samples. These findings show the necessity of considering creep compliance when designing parts subjected to prolonged stress. In assemblies that experience vibrations during use, care should be taken to secure assembly components and prevent unwanted loosening.

It is suspected that the mechanical changes in both PLA and PETG happen because of molecular photodegradation. Such effects have been previously studied at the molecular level by Allen et al., who analyzed the photodegradation of PET under the effect of ultraviolet light [70]. Their work also suggested how homolytic bond scission happens at the start the degradation process, resulting in free radicals that then readily react with oxygen. Their work also suggests that the photooxidation occurs in two phases, namely a rapid phase at the beginning of the process followed by a normal phase. Cheng et al. conducted a similar analysis for PETG copolymers [71] and found similar mechanisms of molecular degradation. Rasselet et al. undertook similar research for PLA [72]. Solutions for mitigating the effect of ultraviolet light on polymers exist in the form of UV stabilization additives that are used to absorb the most damaging parts of the UV spectrum and dissipate the absorbed radiation energy as heat [73,74]. The opposite effect can be reached through the use of photosensitizers to increase the biodegradability of materials [75].

When interpreting the results, one should also take into consideration several process-specific characteristics. For instance, printing process parameters, such as part orientation, infill, and raster angle, have a determining effect on the resulting mechanical properties of a part, as researched in [76] for PLA.

## 5. Conclusions

This study investigated the mechanical changes that occur in two commonly used 3D printing materials, PLA (polylactic acid) and PETG (PolyEthylene Terephthalate Glycol-modified), after they undergo an UV-C exposure cycle. This exposure cycle was meant to simulate what happens to parts after being repeatedly sterilized using ultraviolet radiation.

Following a series of mechanical tests performed on UV-C exposed and non-exposed polymer parts, it was found that extended exposure to radiation weakens mechanical properties. The reduction in mechanical properties (tensile strength, compressive strength) was moderate in PLA parts and very significant (>30%) in PETG parts.

Material creep tests, both tensile and compressive, highlighted the creep effects on the investigated polymers and showed that prolonged exposure to radiation accentuates the negative effects of creep. Because of the reduction in tensile strength caused by radiation, parts subjected to the same level of stress experienced more elongation. In a practical compression creep test, PLA and PETG parts were compressed using a screw and nut assembly tightened at a standard torque. After seven days, the torque needed to untighten the assembly dropped significantly. A bigger drop was observed in parts that underwent accelerated aging through UV-C exposure.

The loss in mechanical properties following irradiation is an element that part designers should account for when designing parts that are meant to be UV-C sterilized. Due to creep characteristics, care should also be taken when designing parts that are meant to be stressed for prolonged periods of time. This includes parts with mechanical compliance and assembled components. The large reduction in mechanical properties found in irradiated PETG parts suggests a different material should be considered. UV-resistant blends from this material are commercially available. Finally, it should be noted that 3D-printing results are heavily influenced by the utilized machine and printing parameters, and technicians should ideally perform in-house mechanical testing before starting production of functional parts.

## Figures and Tables

**Table 1:** 3D printing process parameters.

Material	Nozzle Diameter	Layer Height	Contours	Infill	Infill Pattern	Extrusion Temp.	Bed Temp.
PLA	0.40 mm	0.20 mm	2	100%	Grid 45°/45°	205 °C	45 °C
PETG						235 °C	65 °C

**Table 2:** Dimensional measurements.

	Tensile Creep Specimen		Compression Creep Specimen	
	Width	Height	Y	Z
Nominal [mm]	5.20	3.00	25.20	8.00
PLA Control [mm]	5.204 ± 0.006	3.000 ± 0.004	25.188 ± 0.006	8.076 ± 0.013
PLA UV-C [mm]	5.196 ± 0.005	2.998 ± 0.006	25.194 ± 0.009	8.082 ± 0.007
PETG Control [mm]	5.210 ± 0.008	3.014 ± 0.005	25.280 ± 0.012	7.604 ± 0.004
PETG UV-C [mm]	5.224 ± 0.011	3.006 ± 0.010	25.304 ± 0.017	7.608 ± 0.006

**Table 3:** Tensile strength and stiffness.

Property	PLA (Control)	PLA (UV-C)	PETG (Control)	PETG (UV-C)
Tensile strength	29.54 ± 0.35	26.86 ± 0.35	31.30 ± 0.24	19.48 ± 0.46
Young’s Modulus	2775 ± 26.3	2721 ± 27.7	1648 ± 21.5	1552 ± 11.13

**Table 4:** Compressive strength.

Property	PLA (Control)	PLA (UV-C)	PETG (Control)	PETG (UV-C)
Compressive str.	78.06 ± 0.55	67.69 ± 0.67	65.94 ± 9.0	43.57 ± 0.13

**Table 5:** Loads used for creep testing.

Material	Tensile Properties				Compressive Properties			
	Strength [MPa]	Creep Test [MPa]	Target Load [N]	Applied Load [N]	Strength [MPa]	Torque [N·m]	Applied Load [N]	Creep Test [MPa]
PLA	29.54	7.5	117	12.5 × 9.4	78.06	5.07	2816	40.9
PETG	31.3	8	125	12.5 × 10	65.94			

**Table 6:** Untightening torque test results.

Property	PLA (Control)	PLA (UV-C)	PETG (Control)	PETG (UV-C)
Torque [N·m]	4.92 ± 0.04	4.76 ± 0.04	4.49 ± 0.06	4.10 ± 0.05

## Data Availability

The data that support the findings of this study are available from the corresponding author upon reasonable request.

## Supplementary Materials

The following are available online at https://www.mdpi.com/article/10.3390/polym13244467/s1, Table S1: Measured dimensions of 3D-printed parts under load during tensile creep test, Table S2: ANOVA results for tensile and compressive testing.
